# Experiences from implementation of internet-delivered cognitive behaviour therapy for insomnia in psychiatric health care: a qualitative study applying the NASSS framework

**DOI:** 10.1186/s12913-020-05596-6

**Published:** 2020-08-08

**Authors:** Josefin Kadesjö Banck, Susanne Bernhardsson

**Affiliations:** 1 Region Västra Götaland, Centre for Digital Health, The Unit of ePsychiatry, Sahlgrenska University Hospital, Kastellgatan 1, 413 07 Gothenburg, Sweden; 2 Region Västra Götaland, Research and Development Primary Health Care, Gothenburg, Sweden; 3 Department of Health and Rehabilitation, Unit of Physiotherapy, The Sahlgrenska Academy, Institute of Neuroscience and Physiology, University of Gothenburg, Gothenburg, Sweden

**Keywords:** Insomnia, Internet-delivered cognitive behaviour therapy, Implementation research, NASSS framework, Qualitative study

## Abstract

**Background:**

Insomnia is a common diagnosis among patients in psychiatric health care and effective treatments are highly demanded. Previous research suggests that internet-delivered cognitive behavioural therapy for insomnia (ICBT-i) is helpful for a variety of patients and may be effective for psychiatric health care patients. Little is known about implementation of ICBT-i in psychiatric health care. The aim of this study was to explore experiences among therapists and managers who participated in a pilot implementation of ICBT-i in outpatient psychiatric health care, and to identify determinants for the implementation.

**Methods:**

Semi-structured interviews were conducted with 7 therapists and 5 managers working in outpatient psychiatric health care and directly involved with the pilot implementation. Data were analysed using qualitative content analysis guided by the NASSS framework, combining inductive and deductive approaches.

**Results:**

The analysis revealed 32 facilitators, 21 barriers, and 2 determinants that were both a barrier and a facilitator, organised in 1–5 themes under each of the 7 NASSS domains. Key facilitators included: meeting a demand for treatment options with the ICBT-i programme, the experienced benefits of ICBT-i as a treatment option for insomnia, training and support, engagement and support from managers and the wider system, and a long-term organisation for maintenance of the technology. Key barriers included: low interest in ICBT-i among therapists, difficulty in recruiting patients, perceived low ability in therapists to deliver treatment online, technical problems, and therapists’ competing demands leading to low priority of ICBT-i. Complexity analysis assessed two NASSS domains as simple, four as complicated, and one as complex.

**Conclusions:**

The study contributes new knowledge and insights into the implementation process of ICBT-i in psychiatric health care. Our findings highlight the importance of providing training, support, and guidance in online treatment for therapists when implementing a technological innovation. Technical problems should be minimised and the maintenance and demand-side value for the technology must be clear. Support from managers at all levels is crucial, particularly support to therapists in everyday prioritisation among competing demands. Besides taking the identified determinants into account, managing complexity is important for successful scale-up implementation.

## Background

Insomnia is a psychiatric diagnosis described in DSM-5 [1], with a prevalence of 6–15% in the general population [2]. Insomnia can arise as a primary health issue, where disrupted sleep causes reduced function in daily life for the individual. Insomnia is also observed in high comorbidity with other psychiatric disorders. Eighty to 90% of patients with depression and anxiety, 70% of patients with post-traumatic stress disorder (PTSD), and 91% of patients with previous alcohol problems report significant sleep disruption [3], making insomnia a frequent complaint among patients in psychiatric clinics.

Cognitive behavioural therapy for insomnia (CBT-i) is recommended as a first-line treatment of primary insomnia, as well as for patients with co-morbid depression, anxiety, PTSD, and substance abuse disorders [3]. In the short term, the effect of CBT-i for primary insomnia is comparable with pharmaceutical treatment (benzodiazepine receptor agonists), while in the long term CBT-i has been shown to be more effective than medication [4].

Psychological treatment for mental illness in outpatient psychiatric health care is traditionally delivered face-to-face. However, for about 20 years it has been possible to offer psychological treatment over the internet, often called internet-delivered cognitive behaviour therapy (ICBT) [5]. Important benefits of ICBT include flexibility in time and place for both patients and therapists, and possible cost-effectiveness due to time-efficient administration for the therapists. Studies have shown strong support for ICBT in general, with equivalent treatment effects observed as with face-to-face CBT [6, 7]. The effects of ICBT for insomnia (ICBT-i) have also been found to be comparable with the effects of face-to-face CBT-i [8, 9] and CBT-i delivered as a group treatment [10].

Research on ICBT has generally concerned treatment effects, with less attention paid to the implementation process or to factors which may result in successful implementation [11]. Hindering and facilitating factors at different levels, together termed “determinants”, influence implementation processes and outcomes [12, 13]. The use of a theoretical framework is recommended to improve the understanding of implementation processes and determinants [14]. One such framework is the Non-adoption, Abandonment, and Challenges to the Scale-Up, Spread, and Sustainability of Health and Care Technologies (NASSS) framework [15]. This framework was developed for supporting implementation scale-up of technological innovations. It is based on a synthesis of earlier research into the influence of factors and stakeholders on implementation of technological innovations [16], including earlier published technology innovation frameworks and relevant theories, e.g., the Diffusion of Innovations Theory [17] and the Normalisation Process Theory [18]. The NASSS framework consists of seven domains: 1) the condition, 2) the technology, 3) the value proposition, 4) the adopters, 5) the organisation, 6) the wider system, and 7) embedding and adaptation over time. Each domain can be analysed in terms of its complexity level, which may indicate whether a technology implementation is likely to fail or succeed [19]. Technology-supported programmes characterised by a high degree of complexity in multiple NASSS domains rarely become mainstreamed. The NASSS framework has been used to show how complexities in digitalisation projects can be identified, classified, and used to select appropriate and targeted implementation and scale-up strategies [20, 21].

Previous studies on implementation of ICBT have focused on identifying determinants for implementing the new technology in various health care settings, for various conditions, and in various populations [11, 22–29]. Two studies were conducted in psychiatric health care [11, 29]. Both identified the need for adequate training of therapists and the importance of motivation and support of therapists and their colleagues. To our knowledge, no study has evaluated the implementation process of ICBT-i in psychiatric health care.

Before a new method or technology is implemented in the context of Swedish psychiatric health care, feasibility, barriers and facilitators must be explored. Knowledge on how to facilitate a successful scale-up implementation of ICBT-i in psychiatric health care could be of great value for patients, staff, and the psychiatric health care organisation. The aim of this study was to explore experiences among therapists and managers who participated in a pilot implementation of ICBT-i in outpatient psychiatric health care, and to identify determinants for the implementation using the NASSS framework.

## Methods

### Study design

This study was conducted as part of a process evaluation of a pilot implementation of ICBT-i. An exploratory, qualitative study design was employed, using individual, semi-structured interviews. The NASSS technology implementation framework [15] was used as an analytical framework. This framework was chosen because of its solid theoretical foundation and its focus on the challenges of moving from a small technology project to scale-up, spread and sustainability over time. It was developed for studying complexity and to encourage complex thinking about technological innovations in health care [19] and thus is particularly relevant to apply to the evaluation of this pilot implementation.

The focus of the study was the participants’ experiences of the implementation process, rather than the intervention objectives and treatment outcome. The following research questions were formulated:

- What are the experiences of therapists and managers during implementation of ICBT-i in psychiatric heath care?
- Which barriers do therapists and managers experience during implementation of ICBT-i in psychiatric health care?
- Which facilitators do therapists and managers experience during implementation of ICBT-i in psychiatric health care?

### Context and clinical setting

The study was performed in three outpatient psychiatric health care clinics organised under one university hospital in western Sweden. Two of the clinics are specialised in treatment of adult patients with affective disorders and the third clinic offers outpatient care for substance use disorders. The implementation process was led by a project manager and project colleagues who supported the therapists and managers in the clinics. Pilot clinics were mainly identified by senior managers and to some extent unit managers. ICBT therapists were identified by unit managers at each clinic. The therapists were all certified health care practitioners with previous training and experience in CBT.

### Participants

All therapists and managers directly involved in the implementation were invited to participate in the study. All consented to participate and provided written informed consent. Seven therapists, three unit managers and two senior managers were included. Of the therapists, six were psychologists and one was a social worker. Of the managers, three were psychologists, one was a nurse and one a psychiatrist. All managers were male, and among therapists all but two were female.

The therapists were active in most implementation activities. Unit managers were involved in both practical implementation activities, such as technical administration, and in overall management activities, such as identifying suitable ICBT therapists. Senior managers were involved in their capacity as responsible for the clinics. The senior managers had earlier formed a decision on implementing ICBT in psychiatric health care and this pilot implementation was the first step in that strategy.

### The ICBT-i programme

The ICBT-i programme targeted patients in psychiatric outpatient care with insomnia. Since primary insomnia generally is a condition treated in primary care in Sweden, all patients eligible for this programme suffered from comorbid psychiatric health issues. Patients with psychosis or bipolar disorder were excluded due to contraindication. Patients with primarily somatic or neurologic causes of sleep disturbances, such as sleep apnea and narcolepsy, were also excluded, as well as patients with severe suicide thoughts or other acute conditions.

The ICBT-i programme comprises eight therapy modules and is generally delivered over 8 weeks. Each module contains interventions mediated by text, videos and visualised material, homework exercises, graphs over weekly rating scales, and a messaging system for communication between patient and therapist. The modules are based on the established CBT principles for insomnia [30], are standardised, and can only be adapted to each patient to a certain extent, e.g., by removing modules. The ICBT-i programme was available to patients through an online platform accessed via the established national eHealth service 1177.se, which provided a secure login. All patients were initially assessed in a face-to-face meeting. Progress was measured weekly via clinically validated outcome measures delivered online and personal communication with the patient in text messages within the platform.

### Implementation process

An implementation strategy was developed to prepare the clinics and support the implementation process. The strategy comprised activities such as meetings, technical administration, training and technical support. At each pilot clinic, two to three therapists and the unit manager were involved. Generally, they participated in two preparatory meetings. The meetings aimed to describe ICBT in general and ICBT-i in particular, and to prepare processes such as customising the care routines and incident reporting to each clinic. There were also technical and administrative preparations, such as connecting the clinics to the online platform and distributing access levels. The ICBT-i programme and implementation plan were presented for the whole staff at each clinic, to facilitate their involvement in recruiting patients. Therapists were provided with one full day of training on the programme, the technical platform and ICBT skills. Managers were trained in the technical administration. The implementation activities took place during a period of 2–6 months at each clinic, after which the therapists started their first treatments. Therapists were supervised and supported in clinical and technical issues by an experienced ICBT therapist, initially every second week, and later once per month.

The implementation process was led by a project group consisting of three psychologists, all with prior experience from ICBT. One of them, the first author, had the role of project manager.

### Data collection

Semi-structured interviews were conducted to gather information about the experiences during implementation of ICBT-i. The interviews were conducted as face-to-face dialogues, except one which was made by telephone, at 3–4 months after the therapists started their first ICBT-i treatments. An interview guide with open questions covering the research questions of the study was developed for the study, based on the NASSS framework and focussing on the implementation (ICBT in general and ICBT-i in particular), implementation activities and organisational factors. The interview guide is provided as Additional file 1. The interview guide was reviewed after the first interview, but no changes were considered necessary. The venue was generally the interviewee’s own office. Individual interviews lasted for 13–44 min, were audio recorded, and transcribed verbatim by the first author. The interviews were conducted during May to June 2019.

### Data analysis

Data were collected and analysed using qualitative content analysis, as described by Graneheim and Lundman [31]. The analytical process involved a combination of induction (data-driven analysis) and deduction (theory-driven analysis), also called an abductive approach [32]. Both manifest and latent content was analysed. The analysis was supported by NVivo Version 12 (QSR International Pty Ltd).

First, each interview transcript was read multiple times to obtain a sense of the whole. Using an inductive approach, meaning units relevant for the study aim were identified in the transcripts, condensed, and assigned codes. The first two transcripts were independently coded by both authors and discussed, to provide analyst triangulation and reach consensus on a coding strategy. Next, the NASSS domains served as an analytical framework, and by using deductive principles the codes were categorised as a barrier or facilitator and sorted into one of the seven NASSS domains. Barriers and facilitators were sorted into themes that were developed under each domain. The analysis was led by the first author, with the second author supporting the analytical process and providing verification of content conformity of the categories and themes. Labelling and organisation of the categories were continually checked and modified throughout the analysis in an iterative process.

In a final step of the analysis, the complexity of each domain was assessed and classified in three levels: *simple*, meaning straightforward, predictable, few components; *complicated*, indicating still predictable but multiple interacting components or issues; or *complex*, denoting dynamic, unpredictable, not easily disaggregated into constituent components [19]. In this assessment, findings from user and organisational perspectives, as described in the interviews, as well as the perspectives of the researcher/project manager and what is known from the literature, were combined.

## Results

The analysis identified 32 facilitators and 21 barriers for implementation, and 2 determinants that were both facilitators and barriers. These determinants, categorised and abstracted into 1–4 themes under each of the seven NASSS domains, are presented in Table 1. Complexity of the NASSS domains ranged from simple to complex. Main barriers and facilitators in each theme are summarised below.

**Table 1:** Summary of themes, facilitators, barriers, and complexity level, sorted under the NASSS domains

Domain & complexity level	Theme	Facilitator	Barrier	Facilitator & barrier
**1. The condition** *complicated*	A demand for treatment options	*Psychological treatment missing*		
		Facilitating other treatments		
		Insomnia suitable for online treatment		
**2. The technology** *complicated*	Technical functionality and reliability	Intuitive and satisfactory functionality	Insufficient functionality	
		Stable platform	*Technical problems*	
		*Connected to the national e-health service*		
	Technology features	Providing variation in everyday work	Automatic suicide question	
		Standardisation guarantees quality		
		Clarity and structure		
**3. The value proposition** *simple*	Desirability	*Time and cost effective*		
		*Not time or place bound*		
		Reaching new groups of patients		
	Feasibility for patients in psychiatry	Perceived as effective	Too complicated for patients	
	Secondary benefits	Generally improved mental health		
		Improved treatment competence		
		Improved sleep among therapists		
		Facilitating other digitalisation projects		
**4. The adopters** *complex*	Identifying ICBT-i therapists		*Low interest in ICBT-i*	
			Low availability of therapists	
	Colleagues’ attitudes to ICBT-i	Initial positive attitudes	Ideological differences	
		Attitudes gradually more positive	Scepticism and distancing	
	Recruitment of patients	High demand for ICBT-i	*Few patients identified as suitable*	Low commitment
			*Patients not reached by information*	
	New skills and routines	Less stressful	Communication by text difficult	
		Advantages in communication by text	Non-response to messages stressful	
		High quality treatment	*Disadvantages in online contact*	
**5. The organisation** *complicated*	Managerial engagement	*Support from senior managers*	Concern about negative reactions	
		Engagement in implementation		
	Prioritising ICBT-i		Top-down implementation	
			*Priority issues*	
	Organisational challenges	ICBT-i a solution for organisational challenges	Unclear directives	
			Insufficient digitalisation knowledge	
			Organisational responsibility for insomnia	
	Experiences from implementation activities	Solid and structured		Comprehensive process
		*Hi quality training, supervision and support*		
**6. The wider system** *simple*	Societal development	General societal development		
		Patients’ expectations		
		Pressure from patient organisations		
	Regional support	*Regional strategic goal*		
**7. Embedding and adaptation over time** *complicated*	Challenge to scale up		Fear about long run implications	
			Challenges for future implementation	

*ICBT-i* Internet-delivered cognitive behaviour therapy for insomnia

Key facilitators and barriers are marked in italics

### The condition

#### A demand for treatment options

Participants experienced a lack of psychological treatment options for insomnia in psychiatric health care, and therefore the ICBT-i programme was welcomed. This lack of existing options prevented competition between therapists with different ideological preferences, which facilitated programme implementation.

It was believed that with better sleep, patients would be able to assimilate treatment for their comorbid conditions. ICBT-i was therefore perceived as facilitating treatment for other conditions, something highly demanded by practitioners in psychiatric health care.

*“If you can find an effective way to help patients with it [insomnia] and maybe work with it prophylactically, preparatory or supplementary in relation to other treatments, then I think they think it’s quite good.” (manager)*

### The technology

#### Technical functionality and reliability

The functionality of the platform was generally described as satisfactory and intuitive. Even if some functionality limitations were experienced, participants found the platform to be easy to use compared to other electronic systems. Some participants experienced a need to supplement the system with analogue devices, such as pen and paper, when working with patient material.

Participants generally perceived the online platform used for ICBT-i as stable and reliable. It was also seen as an advantage that the platform was connected to the national eHealth service in Sweden (1177.se).

*“It’s an advantage of course, that it is a national system. That gives greater possibilities, I think, that if problems arise they can be corrected, than if we had invented a system of our own. Because then there are both difficulties in building that type of infrastructure and then having some kind of organisation for software maintenance.” (manager)*

However, technical problems interrupted treatments to different extents, causing some concern and uncertainty for the affected therapists and creating a barrier for implementation.

#### Technology features

Working part time with ICBT-i offered a positive variation in the therapy work. The standardised content of the ICBT-i programme was seen mainly as an advantage because it guaranteed a high minimum level of treatment quality and was less dependent on the therapists’ experiences of working with insomnia or ICBT. Another experienced benefit of the technology was the continuous updates about patient progress and setbacks. This feature helped both the therapist and the patient focus on the agreed issues in their communications and overall treatment. Taken together, these aspects contributed to clarity and treatment structure, which facilitated implementation through favourable experience of the ICBT-i programme. The automatic notifications generated when patients scored high on the weekly question about suicidal thoughts were described as time-consuming rather than helpful, as they often required further suicide risk assessment that was not perceived as necessary.

### The value proposition

#### Desirability

Most participants experienced the time and cost effectiveness of using ICBT-i as a great advantage. Compared to face-to-face therapy, one therapist can treat more patients in less time, providing a clear demand-side value for ICBT-i. A major benefit of the digital format was that ICBT-i is not time or place bound, making it easier for both therapists and patients to engage and adjust the treatment to their everyday routines and other demands. In this way, psychiatric health care could potentially reach patients who otherwise would not have sought care. Participants reported that this made treatment possible and especially well-suited for patients suffering from social phobia or living far from the clinics.

#### Feasibility for patients in psychiatry

Therapists generally experienced ICBT-i as effective for treating insomnia. Patients improved their sleep and reported improvement of their mental health in general, according to the participants. This perceived treatment effect facilitated the implementation process. Some therapists expressed concern that ICBT-i may be too demanding for most patients in psychiatric health care, who often have complex psychological issues.

*“It is, I think, patients who, perhaps due to lack of motivation or depression, are unable to get through this material, even though it’s very well thought out and good and short texts and varied with different media and so on … there are still patients who are unable to make it through.” (therapist)*

#### Secondary benefits

Besides the experienced primary benefits of the implementation of ICBT-i, some secondary benefits were described that further highlighted the overall positive experience of the implementation. Therapists noted an improved general mental health in some patients, not only improved sleep. They also reported that knowledge gained from treating patients with the ICBT-i programme could be applied in face-to-face therapy, and that as a result they had become better skilled in treatment of insomnia regardless of delivery mode.

Therapists even expressed that their own sleeping habits were improved after treating patients using the ICBT-i programme. Managers described that the implementation of ICBT-i gave spin-off effects to other digitalisation projects and made those easier to implement, which made them positive to the ICBT-i implementation.

### The adopters

#### Identifying ICBT-i therapists

Identification of employees at pilot clinics who could learn and work with ICBT-i was not straightforward. Unit managers tried to identify suitable therapists and perceived a lack of interest in working with ICBT-i, despite a general demand for treatment options for insomnia. A few therapists were willing to try ICBT-i, but none were initially deeply interested in the new method, only a few were available to take on this extra task, and only a few had the appropriate competence. These difficulties were barriers to the implementation.

#### Colleagues’ attitudes towards ICBT-i

The attitudes towards ICBT-i as a new treatment for insomnia varied among the participants’ colleagues at the clinics. Some colleagues were immediately positive while others developed more positive attitudes during the implementation process, especially if they observed positive treatment effects. Some questioned ICBT-i. Both managers and therapists pointed out ideological differences as a barrier for implementation. Some colleagues, mainly psychologists trained in psychodynamic therapy, had trouble accepting ICBT-i as a “real” therapy and found ICBT-i to be shallow and insufficient for treating the patients’ problems. This ideological tension manifested itself through distancing from colleagues, and some colleagues even expressed dismay at working with ICBT-i.

#### Recruitment of patients

Some participants described a high demand for ICBT-i from patients and a flow of referrals from colleagues. However, most therapists experienced various difficulties in recruiting patients for ICBT-i, as well as many patients dropping out before programme completion. Some therapists experienced difficulty identifying suitable patients, because patients did not meet inclusion criteria or the treatment was otherwise unsuitable or undesired.

*“I think that if you look at the large number of patients with sleeping problems, you should have been able to get more [patients]. After all, there are a lot of people who have stable conditions, who meet the inclusion criteria and should not be excluded.” (therapist)*

#### New skills and routines

When therapists compared working with ICBT-i to working with face-to-face therapy, both barriers and facilitators emerged. The therapy was experienced as less stressful without the patient in the room. Therapists were more satisfied with their communication with patients through text messages, as the process of writing allowed more time to think about what to proffer. Participants described the ICBT-i programme as appealing, professional, solid, thorough, and educative. However, some therapists found it constraining to communicate with patients in text messages, and expressed that this could lead to misunderstandings and make nuancing statements difficult.

*“I put quite a lot of thought around my messages because I feel that it is difficult to nuance oneself in text. And you still want to be quite concise, warm, affirmative, yet just pushy, which you do not quite know if you are. You know that if you sit eye to eye, you can read a person.” (therapist)*

Not meeting the patient face-to-face was experienced as difficult for the continued assessment, especially regarding patients’ reported suicidal thoughts in the weekly questionnaires, but also for working with motivation or picking up nonverbal signals on patients’ condition, progress or needs.

### The organisation

#### Managerial engagement

The support for implementation of ICBT-i from managers at all levels in the organisation was experienced as strong, primarily due to ICBT-i being aligned with the overall prioritised goal of increasing digitalisation in health care. The active demand for digitalisation activities from the organisation’s upper management created an incentive for change among managers throughout the organisation. Engagement from managers at unit and senior levels was described as essential for successful implementation.

*“I think, the higher up you get, the more you think it sounds like a good thing. And when you are at the unit manager level you think, ‘How on earth should I fit this in with everything else?’.” (manager)*

#### Prioritising ICBT-i

Participants experienced that the implementation of ICBT-i came as a top-down decision, and that the latitude for unit managers and therapists to ignore this decision was limited. ICBT-i implementation was a prioritised project and unit managers were requested to engage their staff. As this did not fully match the existing priorities of therapists or unit managers, this was an implementation barrier.

The ICBT-i implementation was experienced as an additional task on top of everything else. Participants found this priority difficult and the pressure of other assignments, regardless of urgency, was a barrier for identifying ICBT therapists, participating in implementation activities, recruiting patients and starting up new ICBT-i treatments.

#### Organisational challenges

Directives from the organisation concerning implementation of digital solutions in health care in general, and ICBT-i in psychiatric health care in particular, must be clearer, according to participants.

Participants considered the organisation far behind the general digitalisation in society, compared with most other public organisations and authorities, and perceived a lack of digital competence and experience in the organisation.

*“I suspect that there is a range of people, from those who do not at all believe it [ICBT] can work, who think that a face-to-face meeting is needed to achieve something, to those with a gigantic over-belief on digital solutions and its resource-saving possibilities.” (therapist)*

Some participants expressed doubts about whether insomnia is a condition to be treated within psychiatric health care, or if it is rather an assignment for primary care.

#### Experiences from implementation activities

The participants generally experienced the implementation activities and process as being well organised, comprehensive, and well structured.

*“I think the activities have been very good. A clear structure and well thought out. I think this has really facilitated the implementation. It has been realistic to actually be able to do something in the clinic with this.” (manager)*

Participants were satisfied with the content and quality of the training, supervision and support. Therapists expressed that this made it safe and easy to start and follow up the treatments, which facilitated implementation.

### The wider system

#### Societal development

Implementation of ICBT-i was perceived as in line with societal trends on digitalisation and online communication. Patients today expect digital health care that meets their needs, something that could be achieved with ICBT-i. Also, patient organisations are updated on digital options for treatments and communication, and request such alternatives from psychiatric health care. This creates an incentive for change and facilitates the implementation of ICBT-i.

#### Regional support

Participants experienced organisational support for implementation of ICBT-i, not only within their own organisation but also in the regional health authority of which the hospital is a part. For several years, digitalisation has been an explicit strategy to reach the goal of better health in the region’s population. The ICBT-i programme was perceived as an example of digitalisation in line with this strategy and thus received support for the implementation from the wider system.

### Embedding and adaptation over time

#### Challenge to scale up

Some concern was raised about the long-term implications of implementing ICBT-i in psychiatric health care, such as concern that ICBT-i would replace other treatments.

*“If you have been in public health care for a while, you know that money plays a big part. You introduce something as an alternative, and say that this is not going to replace anything. Then you implement it and two years later you remove what you had before.” (manager)*

Participants believed that successful, sustained implementation of ICBT-i in psychiatric health care could be a challenge. Managers pointed out scepticism and lack of knowledge and interest among staff as important barriers to a scale-up. A successful result of this pilot implementation was seen as crucial, but not sufficient, for a successful future scale-up implementation. Because the study was conducted early in the implementation process, with only 3 units participating in this pilot implementation, few findings could be mapped to this domain.

### Complexity

In the condition domain, only facilitators were described, indicating low complexity. However, insomnia is a condition characterised by a high level of comorbidity in the psychiatric population [3], and also influenced by socio-cultural factors, such as being unemployed or having a stressful life [2]. The interview findings, together with general knowledge about insomnia, resulted in the condition domain being assessed as *complicated.*

The technology domain was also categorised as *complicated*. Both facilitators and barriers were described. Although the technical platform used in this implementation is rather well used, tested in many regional health authorities in Sweden, and benefits from a national software maintenance and management structure, the ICBT-i programme has not been evaluated for use in psychiatric health care. Altogether, this domain was assessed as *complicated*.

In the value proposition domain, only facilitators emerged in the interviews. The supply-side value in this case did not complicate the implementation, because the cost for technology development is assigned to the regional health authority, not to the hospital or psychiatric organisation. On the demand-side the participants’ experiences as well as previous studies suggested that ICBT can be effective for insomnia [8], highly cost effective [33], and that attitudes generally are positive [5]. This domain was therefore assessed as *simple*.

In the adopter domain, both facilitators and barriers were described. The barriers indicated that the ICBT-i implementation required a change in professional identity and necessitated difficult judgements, implying a high degree of complexity at the therapy level. At the patient level, ICBT-i was not considered feasible for many of the patients; these patients were excluded and drop-out level was high. Altogether, these challenges for both therapists and patients make the domain *complex*.

In the organisation domain, both facilitators and barriers were identified, and the complexity level was rather high. Participants expressed a negative view of the organisation’s capacity to innovate, based on earlier experiences and implementation projects. However, the engagement from management in the ICBT-i implementation, as well as the highly rated support from an external project group in the implementation activities, reduced the complexity. These aspects resulted in a classification of the domain as *complicated*.

Regarding the wider system domain, only facilitators were described. Since the technology and method is tested and well used nationally [5], possible hurdles in relation to political, financial, legal, regulatory or public concerns have already been managed, making the complexity level of this domain *simple*.

Only barriers were identified in the embedding and adaptation domain. Little is known about how resilient the organisation is regarding adaptation to unforeseen events. One vulnerability in the psychiatric health care organisation for adaptation over time is staff turnover. After the pilot implementation, three of six therapists did not continue working with ICBT-i in the pilot clinics. Based on limited knowledge, this domain was assessed as *complicated.*

## Discussion

In this study, we explored experiences of therapists and managers during a pilot implementation of ICBT-i in outpatient psychiatric health care, and employed the NASSS framework to identify determinants for its implementation related to different domains of the new technology. Most barriers concerned the adopters of the new technology, while most facilitators pertained to the technology’s value proposition and the wider system. In the early implementation process, meeting a demand for treatment options with the ICBT-i programme was experienced as a key facilitator, while the low interest of ICBT-i among therapists was a key barrier. In the continued implementation process, key facilitators were the experienced benefits of ICBT-i as a treatment option for insomnia, training and support, engagement and support from managers and the wider system, and long-term organisation for maintenance of the technology. Key barriers included difficulty in recruiting patients, perceived low ability in therapists to deliver qualitative treatment online, technical problems, and therapists’ competing demands leading to low prioritisation of ICBT-i. These determinants were either explicitly stated by the participants or interpreted as important for the implementation process by the authors from the latent content of the collected data.

The generally low initial interest among therapists in working with ICBT-i was a barrier for providing the method with attention and status of importance in the early implementation process, and for patient recruitment. Difficulties identifying ICBT therapists and recruiting patients, even in the context of limited treatment options for insomnia, implied that implementation was not based on therapist or patient demand. However, interest and attitudes among staff improved during the implementation process, suggesting that this might not be a barrier in the long-term or in a scale-up. Implementation determinants are linked to each other in complex relationships and are likely to vary across different implementation phases and evolve as the implementation process progresses [13]. As proposed in Roger’s Diffusion of Innovation theory [17], innovations are not adopted by all individuals in a social system at the same time. With positive experiences from early adopters, the ICBT-i method is likely to, with time, spread to the majority of therapists and their managers.

Difficulty recruiting patients was also identified as a barrier in a previous study on implementation of ICBT in psychiatric health care [11]. To address these difficulties, it may be useful to look at the compatibility between the ICBT-i programme and the patients. For example, it may be that the inclusion criteria should be narrower. Finding suitable patients that may benefit from ICBT-i is likely to be more challenging in psychiatric health care than in primary care, due to patients suffering from more severe mental illness. Increased colleague engagement is also important for recruitment, as well as stable routines for internal referral and communication among therapists. Generally, patients seem to be more positive to ICBT than clinicians [5], and patient demand for this technology might increase with adequate information.

The concerns about treatment quality, expressed mainly by colleagues of the ICBT therapists, have also in previous research been shown to be a barrier for successful technology implementation [11, 26, 27, 29]. In our study this was the case especially in clinics characterised by disagreement over the most appropriate therapeutic method. This may be related to the classic debate regarding psychodynamic therapy versus CBT, rather than implementation of the internet-delivered version of CBT-i. To enable a scale-up implementation of ICBT-i, it may be necessary for managers to review the general CBT competence among therapists in psychiatric health care and to consider this in future recruitment. Treatment quality concerns can be addressed through continuous assessments of the treatment effects of ICBT-i, which have not yet been sufficiently investigated in patients in psychiatric health care.

The need for new skills related to the shift from face-to-face to online therapy has been highlighted as a barrier in a previous study, also conducted in psychiatric health care [29]. To secure treatment quality and therapeutic confidence, this need must be addressed in the training and supervision of new ICBT therapists.

Support from senior managers was experienced as crucial and a key organisational facilitator that motivated therapists and facilitated unit managers to prioritise implementation of ICBT-i. Organisational support and leadership are well known facilitators and among the most common contextual determinants that may facilitate implementation [12]. Leadership engagement and management support were highlighted in a recent review as important facilitators for effective implementation of a wide range of e-health innovations [34]. By clearly prioritising ICBT-i, senior managers paved the way for a smooth first phase of the implementation. This prioritisation by senior managers was enabled by the regional health authority’s clear strategy to prioritise digitalisation projects for improving public health. However, despite this, in therapists’ everyday practice, unresolved priority issues formed organisational barriers to the programme’s implementation. Previous studies have highlighted competing demands to the ICBT treatments, especially regarding available time for the therapists to engage in ICBT treatments [22, 26–28], implying that therapists need protected time for ICBT-i. This barrier also indicates that implementing ICBT in clinics or organisations with a wider health care assignment requires other strategies than in specific ICBT clinics.

The technology itself was described as generally reliable and functional, but technical problems that arose during treatments affected the therapists’ trust in the technology. However, the connection to the national eHealth service was an important facilitator because participants found this to guarantee security for patients and also to ensure maintenance of the system. Technical problems have also been identified as a barrier in previous studies [22, 23]. Some technical obstacles were identified during the pilot implementation, and for a scale-up implementation it would be of utmost importance that these technical problems and uncertainties are minimised. Some issues interpreted by participants as technical problems might have been the therapists’ lack of knowledge, further indicating the importance of technical support and supervision and suggesting a need for more real-life training, which may be achieved by a larger-scale recruitment of patients.

The experienced treatment effects of ICBT-i are supported by previous research [6]. Benefits such as accessibility and flexibility for the patient regarding time and place of treatment were highlighted in a previous study on preference for ICBT [24]. The experienced benefits are consistent with the major attributes of an innovation that Rogers [17] proposed; notably the relative advantage of the new technology over previously used methods and observability of results. Trialability, compatibility and complexity of the technology also contribute to the benefits of ICBT-i. In times of limited resources and an increasing need for psychiatric health care, the identified benefits of ICBT-i will be motivating for diverse stakeholders in a scale-up implementation.

The finding that implementation activities, such as therapist training and supervision, facilitated the implementation, is also supported by previous studies. Training regarding the evidence base for internet-delivered treatment has been shown to facilitate ICBT implementation [11, 26–28], as well as practical training in the programme itself [26], supervision of therapists during the intervention [11, 28], and technical support [25]. In a scale-up implementation it is important to meet the continuous need for training, supervision, and support of the ICBT therapists.

### Methodological considerations

The use of an implementation framework contributed to systematic data collection and analysis, which can increase credibility of the findings [35]. The complex thinking of technological innovations in health care, encouraged by the NASSS framework, provides the opportunity to highlight complexities that with careful handling can contribute and enable a successful implementation, with scale-up, spread and sustainability over time.

The sample was rather small and homogeneous in terms of the participants’ profession. Other perspectives could have provided valuable insights for a scale-up implementation. It is therefore a limitation that we did not include first-hand perspectives from patients, colleagues of the participating therapists, and representatives of the wider system, such as political decision makers. Another limitation is that the first author was both project manager for the implementation, conducted the interviews, and led the analysis. Her knowledge of the project and relation to the participants might have affected the interviews, analysis, and interpretation of the results. On the other hand, familiarity with the context can be a valuable asset and contribute to deeper discussions in the interviews, as well as a more complete understanding in the complexity analysis.

### Implications for practice and research

The identified implementation determinants, as well as complexity levels of different domains of the technological innovation, provide valuable insights for policy makers, managers, and health care providers and can help guide implementation and scale-up. The barriers and facilitators experienced by therapists and managers in this ICBT-i pilot implementation must be met for a successful implementation. Our findings highlight the importance of providing training, support, and guidance in online treatment to therapists when implementing a technological innovation. Technical problems must be minimised, and the maintenance and demand-side value of the technology should be clear. Support from managers at all levels is crucial, particularly support to therapists in everyday prioritisation among competing demands. The findings of this study may be transferable to other clinics and hospitals implementing ICBT as a treatment option in psychiatric health care and may be of special interest for organisations where staff lack experience from internet-delivered therapy or ICBT.

This study also adds to the growing information available on the NASSS framework, by providing an example of how the framework supported the categorisation of challenges experienced during the pilot implementation of a new technology and how a complexity analysis can be used to develop an implementation strategy for scaling up pilot implementations. The NASSS complexity analysis in this study indicated that the benefits of ICBT-i are considerable for the organisation, therapists, and patients. Support from the wider system facilitated the pilot implementation and could possibly alleviate the barriers within the system in a successful scale-up implementation. The identified determinants and the complexity levels of the different domains must be considered when designing a strategy for scaling up ICBT-i implementation. In this study, the greatest barriers for a successful scale-up of ICBT-i were within the adopters domain. It may be possible to reduce complexity of the adopters domain, among other domains, to facilitate scale-up. However, as the programme and the technology evolve, the complexity can be expected to increase or decline, further underscoring the need to address complexity in scale-up strategies, and maybe revise strategies along the road.

For future implementation of ICBT-i in psychiatric health care it will be important to not only assess the implementation process but to also investigate the effectiveness of ICBT-i treatment for patients with severe mental illness, an area not yet widely explored.

## Conclusions

This study shares the experiences of therapists and managers from participating in a pilot implementation of ICBT-i in outpatient psychiatric health care. Our findings contribute new knowledge about the implementation process and about determinants for implementation of a complex technology in a health care setting. The identified barriers and facilitators, as well as complexity levels of different domains of the technological innovation, provide valuable insights for policy makers, managers, and health care providers to guide implementation and scale-up.

The key barriers identified in this pilot implementation were to a high degree related to the adopters of the innovation, such as low interest in ICBT-i from therapists and difficulties in recruiting patients. These findings underscore the importance of addressing such hindering factors in a scale-up implementation. The key facilitators identified in this study indicated that stakeholder support at different organisational levels and a clear demand-side value are needed for a successful implementation and are similarly important for a successful scale-up.

With careful management of complexity when scaling up implementation, ICBT-i has the potential to be successfully implemented in psychiatric health care. Above all, managing complexity related to the adopters of the technology is important for succeeding in a scale-up implementation, but complexities related to the condition, the technology, the organisation, and embedding and adaptation over time, must also be managed.

## Supplementary information

**Additional file 1.** Interview guide.

## Data Availability

The interview transcripts are available from the authors upon reasonable request.

## Abbreviations

NASSS: Non-adoption, Abandonment, and Challenges to the Scale-Up, Spread, and Sustainability of Health and Care Technologies
CBT: Cognitive Behavioural Therapy
ICBT: Internet-delivered Cognitive Behavioural Therapy
ICBT-i: Internet-delivered Cognitive Behavioural Therapy for Insomnia
