# Supplementary material for: Experiences from implementation of internet-delivered cognitive behaviour therapy for insomnia in psychiatric health care: a qualitative study applying the NASSS framework
Source: BMC Health Serv Res. 2020 Aug 8;20:729. doi: 10.1186/s12913-020-05596-6 (PMC7414663; doi:10.1186/s12913-020-05596-6)
Supplement: Supplementary file 1 — Additional file 1. Interview guide. [file 12913_2020_5596_MOESM1_ESM.docx]

# **Additional file 1. Interview guide – implementation of iCBT-i**

**The implementation object** *Domains: the techology, the value proposition, adopters, the condition*

- What advantages and drawbacks have you experienced when treating patients with insomnia with ICBT-i?
- How have you experienced the attittude toward ICBT-i among your colleagues at the clinic? (Have you experienced that the attitudes have changed during the course of the implementation?)
- (How do you perceive the need for effective treatments for insomnia at your clinic?)
- How have you experienced the treatment to work for the patients; how have they been helped?
- How have you experienced the digital systems you’ve been involved in during the implementation?
  - For treatment
  - For implementation/administration

**Organisational factors** *Domains: the organisation, the wider context*

Therapist:

- How have you been able to prioritise the work with ICBT-i in relation to other tasks?

Manager:

- What possibilities have your staff had to prioritise working with ICBT-i in addition to their other tasks?
- How do you perceive that the hospital as an organisation views implementation of this type of treatment (ICBT-i) in psychiatric care?

**Implementation activities** *Domains:* *Embedding over time, the technology*

- What are your experiences of the activities that were part of the implementation? (meetings, emails, guidance/manuals, training, tech support)
- Have you, as therapist/manager, received the information and support you needed in conjunction with the implementation?
  - If no – what was missing?
  - If yes – what type of support have you experienced as especially useful?

**Additional questions** to tease out more information about barriers and facilitators:

- Can you think of any particular factors that you feel were barriers for the implementation of ICBT-i at your clinic?
- Can you think of any particular factors that you feel were facilitators for the implementation of ICBT-i at your clinic?
